# Supplementary material for: The role of race and insurance in trauma patients’ mortality: A cross-sectional analysis based on a nationwide sample
Source: PLoS One. 2024 Feb 15;19(2):e0298886. doi: 10.1371/journal.pone.0298886 (PMC10868734; doi:10.1371/journal.pone.0298886)
Supplement: S1 Appendix — (DOCX) [file pone.0298886.s002.docx]

Appendix 1. Sensitivity analysis

Overall group in-hospital mortality multivariate logistic regression analysis with interaction term

* ACAME = Affordable Care Act Medicaid Expansion

Short LOS group in-hospital mortality multivariate logistic regression analysis with interaction term

* ACAME = Affordable Care Act Medicaid Expansion

Long LOS group in-hospital mortality multivariate logistic regression analysis with interaction term

* ACAME = Affordable Care Act Medicaid Expansion
