# Supplementary material for: The role of race and insurance in trauma patients’ mortality: A cross-sectional analysis based on a nationwide sample
Source: PLoS One. 2024 Feb 15;19(2):e0298886. doi: 10.1371/journal.pone.0298886 (PMC10868734; doi:10.1371/journal.pone.0298886)
Supplement: Attachment — Submitted filename: REVIEW OF role of race and insurance in trauma patients mortality a cross sectional analysis based a nationwide sample .docx [file pone.0298886.s003.docx]

REVIEW OF “***THE ROLE OF RACE AND INSURANCE IN TRAUMA PATIENTS’ MORTALITY: A CROSS-SECTIONAL ANALYSIS BASED A NATIONWIDE SAMPLE***

The study examines the effect of insurance status, race, and length of hospital stay on inpatient mortality. The study has several points of strength and weakness. All the points that need to be reviewed are discussed section by section as well as the specific location of text in the study.

## Abstract

The abstract needs to be reviewed for the clarity of the study question, inclusion and exclusion criteria, and interpretation of the study findings. It should also be reviewed for the redundant text especially when it mentions the effect of race on the findings. The study did not use uniform terminologies throughout the paper e.g., inpatient mortality mentioned in the abstract is referred to as in-hospital mortality in the data table 2.

## Introduction

This section explains the previous research done on the topic and reveals the gaps that are to be filled in the current study. However, it fails to convince the reader that in the presence of EMTALA, the trauma patient at extreme risk of mortality with no insurance coverage is at a disadvantage. According to the EMTALA (Emergency Medical Treatment and Labor Act), all patients requiring emergency medical treatment have the right to be treated or stabilized regardless of their insurance status.

## Method

This section explains the study design, study settings, and the data source. It is a lengthy section with multiple subheadings with each subheading explaining redundant and incoherent information that can be confusing to the reader. E.g., the “study design” section explains the data source, and then in “Settings” the data source is mentioned again.

The subheading “participants” is inappropriate as the study is performed on the secondary source data and the patient information used in the study is already deidentified to protect confidentiality. Consent for using the data source and statement of Conflict of interest for mentioning a commercial product should also be mentioned. In the subheading “bias” there is an unnecessary explanation of the NIS methodology for data collection and its evolution over the years. There is no mention of the version of APR-DRG used.

## Quantitative variables:

The section presents redundant, incoherent information that can be reviewed for clarity. the term LOS throughout the paper has been defined multiple times but at each time the definition is non-homogenous and confusing. Here LOS is defined as early and late groups with the early group having been discharged within two days and the late group with LOS greater than 2 days. Later in the paper, there is mention of 2 groups with LOS of 2 days and others with LOS of more than 2 days without mention of the term “early or late”

## Statistical method:

This section relies on the use of approximations e.g., (analysis used a combination of multiple imputation and survey data analysis modules in Stata) and does not clearly mention the specific statistical tests done to control for the missing data like e.g. (expectation maximization method), survival treatment assignment bias (cox regression, Kaplan Meier survival analysis, etc.).

## Results:

This section has multiple erroneous data interpretations. LOS here refers to 2 days for one group and more than 2 for another group unlike what has been defined earlier. The interpretation of the racial variable for Table 2 is erroneous.

## Discussion:

The discussion is detailed but lacks practical implications of the findings and how they can influence future research. There is a big contradiction in the discussion part where it says that the pattern of LOS and mortality in African Americans is similar to Hispanics and other races which is an incorrect interpretation of the analysis. The study refers to several other studies that found racial disparities in health outcomes but failed to include race-specific insurance data for the sample in the analysis. Had race-specific insurance data been analyzed it would have demonstrated the relation between racial disparity in health insurance and subsequent care.

## Specific comments:

Line 26: “70,381 discharges” is a confusing term, it would be clearer if it is substituted by the in-hospital mortality

Line 75: the hospital participating to NIS should be reviewed to match the up-to-date information

Line 80: which version of APRDRG is used?

Line 116: The timing of the insurance coverage does not matter in this condition as the patient is covered by EMTALA

Line 129: this statement does not make sense as the study participants are 65 or younger

Line 133/134: Confusing

Line 143/line 154: there should be uniformity in using terminology line 143 says all community hospitals except long-term care facilities while line 154 says excluding govt institutions where whereas NIS data comprised of all us community hospitals except the long-term care and rehabilitation. This sample definition should be consistent with the NIS 2020 definition. <https://hcup-us.ahrq.gov/nisoverview.jsp>

Line 147: What were the confounding factors and which statistical analysis was performed to control it.

Line 148 what is meant by survey data analysis modules ??? specify the statistical test used for the study data

Line 155: MCAR (missing completely at random) analysis should be done and the p-value should be mentioned in order to satisfy the assumption.

Line 157: Please specifically mention the statistical test performed for data imputation e.g. expectation maximization method.

Line 177: there is an error in the interpretation

LINE 191: Again, there is nonuniformity in defining the LOS, it is mentioned LOS is 2 days for one category and more than 2 days for another category while initially it was mentioned as 2 groups LOS within 2 days and LOS with 2 days

Line 192: again, the LOS is defined in a different manner

Line 219: LOS is mentioned as a short stay (within 2 days) and long stay, unlike earlier where it is mentioned as early and late

Line 231: 26-50^th^ income percentile has the same effect as 76^th^ to 100^th^ income percentile

Line 256: Hispanic and “other” races show the reverse of the pattern shown by African Americans

## Decision:

The study should undergo major revision for their methodology, usage, and interpretation of the data. The findings should be presented with clarity and accuracy to draw logical conclusions from the study question. The terminologies used in the study are inconsistent and should be standardized. It seems that the author is completely unaware of the EMTALA act according to which all patients coming to ER for emergency treatment will be treated immediately and equally regardless of the insurance status. The inference drawn from this study can be misleading to the reader. The mortality may just be related to the severity of the initial trauma. APR-DRG system provides data for the severity of illness, which if considered in the analysis can give a better estimation of the outcome for such patients.
