# Supplementary material for: The role of race and insurance in trauma patients’ mortality: A cross-sectional analysis based on a nationwide sample
Source: PLoS One. 2024 Feb 15;19(2):e0298886. doi: 10.1371/journal.pone.0298886 (PMC10868734; doi:10.1371/journal.pone.0298886)
Supplement: Attachment — Submitted filename: Rebuttal Letter.docx [file pone.0298886.s004.docx]

Dear Academic Editor and Reviewers,

I express my deepest gratitude for the extensive and meticulous review of my manuscript, along with the invaluable feedback that has been provided. Your observations and detailed critiques were invaluable.

The abstract has undergone extensive revision for enhanced clarity, precise definition of the study question, and clear articulation of inclusion and exclusion criteria. Redundant text, especially concerning the effects of race, has been removed. Priority was given to standardizing the terminology across all sections of the paper (e.g., inpatient mortality vs. in-hospital mortality).

I expanded the literature review, addressing the relationship between insurance status, race, and outcomes in trauma patients, and I elaborated on the varied interpretations and conclusions drawn in previous studies. Additionally, I incorporated a discussion on the Emergency Medical Treatment and Labor Act (EMTALA), examining its implications on this study and its role in shaping patient outcomes.

I revised the entire methods section for brevity, when necessary. I elaborated on the consent exemption process, as well as any potential conflicts of interest. Regarding statistical analysis, I now provide specific details on the tests used and the rationale behind their selection.

Concerning variables associated with length of stay and race in the results section, I fully recognize the mistakes made in my initial interpretation, and I worked to correct these errors to present a more accurate portrayal of the data.

Lastly, I revised the discussion section to elucidate the practical implications of the findings, creating a stronger link between theory and practical application. I also note opportunities for future research.

Specific comments:

**Line 26: “70,381 discharges” is a confusing term, it would be clearer if it is substituted by the in-hospital mortality**

Thank you for this comment. I understand that the term “discharges” might seem ambiguous, especially when considered within the context of hospital mortality rates. However, as outlined by the National Inpatient Sample (NIS; https://hcup-us.ahrq.gov/db/nation/nis/nischecklist.jsp), the primary unit of analysis in the NIS pertains to inpatient stays rather than individual patients. Hence, “70,381 discharges” denotes the cumulative number of inpatient stays within the sample study, rather than the number of in-hospital mortality incidents. Nonetheless, I revised to “stays” in the text to prevent potential misunderstandings for readers unfamiliar with the NIS terminology.

**Line 75: the hospital participating to NIS should be reviewed to match the up-to-date information**

Thank you for this comment. This dataset encompasses inpatient discharges from community hospitals in 48 states and Washington DC. By definition, community hospitals include all non-federal, short-term, general, and other specialty hospitals, excluding hospital units of institutions. I amended the manuscript to accurately reflect the current status of hospitals participating in the NIS.

**Line 80: which version of APRDRG is used?**

Thank you for this question. I amended the manuscript to reflect that the APRDRG versions were updated annually during the course of the study years.

**Line 116: The timing of the insurance coverage does not matter in this condition as the patient is covered by EMTALA**

Thank you for pointing out the relevance of EMTALA in the context of emergency care. You are right that EMTALA ensures that every individual is evaluated and stabilized in emergency situations irrespective of their insurance status. However, while EMTALA mandates the provision of emergency care to all, regardless of insurance status, it does not ensure equity in the quality or continuity of subsequent care. Especially for trauma patients at an extreme risk of mortality, the post-stabilization care, which is crucial for their survival and recovery, might be influenced by their insurance coverage and other socio-economic factors. In addition, compliance with the mandate is not necessarily equal across institutions. Nonetheless, including this issue in the manuscript is very relevant, so I revised accordingly.

**Line 129: this statement does not make sense as the study participants are 65 or younger**

Thank you for bringing this to my attention. You are correct that Medicare typically covers individuals aged 65 years or older. However, it is worth noting that certain younger individuals with disabilities or specific diseases (e.g., end-stage renal disease or ALS) can also be eligible for Medicare benefits. In the NIS dataset, the categorization of insurance type is based on the information provided. While most participants are aged ≥ 65 years, there might be a small subset of younger patients with conditions that make them eligible for Medicare. Nevertheless, I acknowledge that this can cause confusion for the readers. For clarity, I amended the manuscript to reflect the above.

**Line 133/134: Confusing**

I revised as follows for additional clarity: “Assessing the impact of hospital stay duration on in-hospital mortality involved categorizing duration into two groups based on length of stay (LOS): short LOS, comprising stays less than two days; and long LOS, encompassing stays of two days or more.”

**Line 143/line 154: there should be uniformity in using terminology line 143 says all community hospitals except long-term care facilities while line 154 says excluding govt institutions where whereas NIS data comprised of all us community hospitals except the long-term care and rehabilitation. This sample definition should be consistent with the NIS 2020 definition.**

Thank you for highlighting this inconsistency. I ensured the manuscript aligns with the NIS 2020 definition. Specifically, I revised the description to reflect that community hospitals, per the NIS definition, comprise “all non-federal, short-term, general, and other specialty hospitals, excluding hospital units of institutions.” This encompasses not only specialty hospitals, such as those focusing on obstetrics-gynecology, ear-nose-throat, orthopedic, and pediatric care, but also includes public hospitals and academic medical centers.

**Line 147: What were the confounding factors and which statistical analysis was performed to control it.**

Thank you for your query regarding confounding factors. In this study, I identified age, race, median household income, sex, and length of stay as potential confounders that could influence the relationship between the predictor variable—insurance—and the outcome. To effectively control these potential confounders and more accurately gauge the impact of insurance on the outcome, I employed multivariable regression analyses. In addition, I updated the sensitivity analysis to include an interaction term between insurance and race.

**Line 148 what is meant by survey data analysis modules ??? specify the statistical test used for the study data**

“Survey data analysis modules” refers to specialized statistical techniques and software functionalities tailored for analyzing complex survey data. Survey datasets, like the one derived from the NIS, have a complex design that includes stratification, clustering, and weighting. In this analysis, I employed Stata’s svy command to account for the complex survey design inherent in the NIS dataset. By using the svy prefix before the statistical analyses, I ensured accurate standard error estimations, which provide more reliable results than traditional methods that do not consider these survey complexities. In the study I employed multivariate logistic regression analyses, adjusted for the NIS’s complex survey design, to analyze the data.

**Line 155: MCAR (missing completely at random) analysis should be done and the p-value should be mentioned in order to satisfy the assumption.**

I apologize for this oversight. In the original manuscript, the missing data assumption was not MCAR but instead was missing at random (MAR). While the data may not meet the MCAR assumption, it is likely to satisfy the MAR assumption.

**Line 157: Please specifically mention the statistical test performed for data imputation e.g. expectation maximization method.**

Thank you for this comment. To address missing race data, multiple imputation via chained equations was employed, utilizing multinomial logistic regression analyses to accommodate the categorical nature of the race variable.

**Line 177: there is an error in the interpretation**

I acknowledge the error and made the necessary changes.

**Line 191: Again, there is nonuniformity in defining the LOS, it is mentioned LOS is 2 days for one category and more than 2 days for another category while initially it was mentioned as 2 groups LOS within 2 days and LOS with 2 days & Line 192: again, the LOS is defined in a different manner & Line 219: LOS is mentioned as a short stay (within 2 days) and long stay, unlike earlier where it is mentioned as early and late**

I apologize for the inconsistency. I revised to “short LOS” and “long LOS” throughout the manuscript.

**Line 231: 26-50th income percentile has the same effect as 76th to 100th income percentile**

Thank you. I revised accordingly.

**Line 256: Hispanic and “other” races show the reverse of the pattern shown by African Americans**

I revised this section to accurately reflect the data, addressing the patterns exhibited by different racial groups. I also made the following revisions for additional clarity:

1) I deleted Tables 2 and 3 from the original manuscript
2) I include an appendix with data from the sensitivity analysis
3) I revised the tables to ensure uniform length of stay data
4) I removed excess subtitles from the methods and results sections

I am dedicated to improving my manuscript to meet the high standards of professional scholarly communication and appreciate the guidance given to help me reach that goal. Please let me know if further revisions are required.

Sincerely,

José A. Acosta MD, MBA, MPH
