# Supplementary material for: The role of race and insurance in trauma patients’ mortality: A cross-sectional analysis based on a nationwide sample
Source: PLoS One. 2024 Feb 15;19(2):e0298886. doi: 10.1371/journal.pone.0298886 (PMC10868734; doi:10.1371/journal.pone.0298886)
Supplement: Attachment — Submitted filename: Response to Reviewers.docx [file pone.0298886.s005.docx]

January 14, 2024

Souparno Mitra, MD

Academic Editor PLOS ONE

Re: The role of race and insurance in trauma patients’ mortality: A cross-sectional analysis based on a nationwide sample

Dear Dr. Souparno Mitra

I am writing to express my sincere gratitude for the time and effort dedicated by you and the reviewers in evaluating the manuscript. The insightful comments and constructive feedback provided have been invaluable in enhancing the overall quality and clarity of this study. I appreciate the thoroughness of the review process, which has significantly contributed to refining this project and ensuring its alignment with the high standards of PLOS ONE.

I have carefully considered and addressed each comment raised by the reviewers, and below, I detail the revisions and clarifications made in response to their valuable feedback.

**Reviewer #3: Introduction:**

**General comment: The Introduction is too brief; it could have elaborated more on the existing data on knowledge gaps on the topic.**

Thank you for your thorough review and constructive feedback on this manuscript. Your suggestion to expand the introduction has been invaluable. In response, I have revised and extended this section to address the existing knowledge gaps more comprehensively regarding the interaction between insurance, race, and in-hospital mortality in trauma patients at extreme risk of mortality.

The revised introduction now includes additional context and references to emphasize the current state of research in this area. These enhancements aim to clarify the specific gaps this study seeks to address and articulate the relevance of this research. The revisions also improve the flow and coherence between paragraphs, ensuring a smoother transition of ideas for the reader. I believe these changes substantially enrich the manuscript and provide a clearer framework for understanding the significance of this study's contributions.

**Line 68-69: Add reference for the statement "However, while this act guarantees the doorway to immediate medical attention, it stops short of ensuring a uniform quality or continuity of care in the post-stabilization phase."**

References have been added to the above statement in the introduction.

**Methods:**

**Line 79-80: Please elaborate this statement to make it clearer "The NIS represents 35–39 million annual hospitalizations from 7–8 million raw hospital stays". This will also help in clarifying statements in the Result section (line 126-130) like, "Among these aged 18–64 years, 715,368 inpatient stays met the criteria for trauma, corresponding to a national estimate of 3,512,361. Of these 70,381 had EROM, which correlated with a national estimate of 346,659 stays, which serve as the basis for this study."**

I appreciate the reviewers highlighting an area of the manuscript which lacks clarity. The National Inpatient Sample (NIS) is a large publicly available database which uses a complex survey design allowing for the creation of national estimates. It is notable for its use of weighted estimates. Each record in the database comes with a discharge weight, compensating for the sampling design and the probability of hospital selection. Applying these weights in statistical analysis allows researchers to extrapolate findings to the national level, making the results broadly applicable to most of the U.S. hospital discharge population.

The data reported in the NIS is based on hospitalizations and not unique individuals. In other words, individual observations are hospitalizations events rather than unique patients [1]. In order to account for this and improve readability the following statement was added to the manuscript. “In the NIS, each record represents a hospitalization event, not a unique patient [1]. For this study, to simplify statistical analysis and interpretation, each hospitalization is considered and referred to as a separate patient encounter. This approach is applied even in cases where individuals may have more than one admission for the same clinical issue.” The manuscript has been revised to consistently refer to 'patients' instead of 'hospitalizations' for improved clarity and coherence.

**Line 107: change "handled" to handle"**

Thank you for pointing this out. The manuscript has been corrected.

**Results:**

**Line 127: Please elaborate the units of 506,403,411 (hospitalizations).**

Thank you for this inquiry. The manuscript has been revised to consistently refer to 'patients' instead of 'hospitalizations' for improved clarity and coherence. Please see above in the response to the Methods section comments for more detailed explanation.

**Discussion:**

**Line 229-238: Another limitation that I can see is that you have not stratified the results before and after Affordable Care Act came into force in 2014. This Act improved access to affordable insurance. This stratification could have highlighted any differences in outcomes before 2014 (when less people were insured). Your study had very few uninsured patients.**

Thank you for the valuable suggestion regarding the stratification of results in relation to the Affordable Care Act Medicaid Expansion (ACAME) in 2014. In response, I have incorporated an ACAME covariate into the multivariate logistic regression model and updated the results accordingly. These changes are now reflected in Table 2 and have been discussed in the revised manuscript. I am grateful for this insightful recommendation.

**Reviewer #4:**

I am grateful for the comprehensive review and insightful feedback provided reviewer #4. The reviewer’s acknowledgment of the significance of this research topic is greatly appreciated.

In conclusion, the manuscript has been revised and updated in response to the valuable feedback received. The modifications made address the comments and suggestions enhancing the clarity, and overall quality of the study. I welcome any further suggestions or comments to improve the manuscript. Thank you for considering my revised manuscript for publication in PLOS ONE. I am hopeful that the revisions meet the high standards of your journal.

Sincerely,

José A. Acosta MD, MBA, MPH

1.	Khera R, Angraal S, Couch T, Welsh JW, Nallamothu BK, Girotra S, et al. Adherence to methodological standards in research using the national inpatient sample. JAMA. 2017;318(20):2011-8.
